# Synergistic enhancement of zinc anode stability via bismuth alloying and CO_2_ exposure in 1 M KOH for alkaline battery applications

**DOI:** 10.1038/s41598-026-52415-9

**Published:** 2026-05-21

**Authors:** Mostafa Adel, Abdelrahman Elsayed, Mahmoud Elrouby

**Affiliations:** 1 Chemistry Department, Faculty of Science, Sohag University, Sohag, 82524 Egypt; 2 Faculty of Science, Sohag National University, New Sohag, Sohag, 82511 Egypt

**Keywords:** Zinc–bismuth alloys, KOH media, Corrosion inhibition, Hydrogen evolution suppression, CO₂ tolerance, Electrochemical stability, Chemistry, Energy science and technology, Materials science

## Abstract

This study systematically investigates the electrochemical performance of pure zinc and zinc-0.5 wt% bismuth electrodes employed as anodes in alkaline battery systems using a 1 M KOH electrolyte, under both CO₂-free and CO₂-saturated environments. Electrochemical behavior was evaluated through potentiodynamic polarization, electrochemical impedance spectroscopy (EIS), and galvanostatic charge–discharge cycling. Surface morphology, elemental composition, and phase structure were analyzed using scanning electron microscopy coupled with energy-dispersive X-ray spectroscopy (SEM-EDX) and X-ray diffraction (XRD). The introduction of CO₂ markedly reduced the corrosion current density (*i*_corr_) of both electrodes, indicating a significant mitigation of corrosion processes. Notably, the zinc–bismuth alloy exhibited superior electrochemical performance compared to pure zinc. Under CO₂-containing conditions at 25 °C, the Zn-Bi electrode achieved a maximum corrosion inhibition efficiency of 87.615%. Galvanostatic cycling results further demonstrated enhanced charge–discharge behavior, improved capacity retention, and greater electrochemical stability in the presence of CO₂. These results highlight the strong potential of Zn-Bi alloys as durable and high-performance anode materials for advanced alkaline battery applications.

## Introduction

Zinc-ion batteries (ZIBs) represent a promising alternative to lithium-ion systems. They offer comparable energy and power densities with better economic viability. ZIBs provide improved safety features and environmental compatibility. These characteristics make them attractive for next-generation energy storage applications^1–8^. The low reversibility of Zn plating/stripping at the Zn anode|electrolyte interface is caused by zinc dissolution, dendritic development with shape change, hydrogen evolution reaction (HER), corrosion, and passivation, which makes ZABs impractical. The reversibility of Zn plating/stripping can be enhanced by the combined computational and experimental studies using a number of tactics meant to overcome these obstacles. Verifying the results of computational research usually involves techniques like anode structure optimization, electrolyte composition tweaking, and interface engineering^9^. Nevertheless, the dendritic development on the zinc surface and HER remain two glaringly harmful barriers in the use of ZIBs at scale. This significantly reduces the cycling stability and Coulombic performance of ZIBs^10,11^. It is necessary to take steps to inhibit HER since it affects ZIB performance by lowering (CE), which causes cell expansion and Zn anode corrosion^12,13^. There is a strong association between HER and dendritic growth since both are impacted by the anode’s surface condition and the contact between the electrolyte and anode. Dendrite development, for instance, will produce a porous anode with a greater number of surface area reaction sites, which will speed up HER^14^. Consequently, if one problem is resolved, the other will also be lessened. The HER may usually be decreased by modifying the electrolyte, the anode, and their contact^15–22^. As an electrolyte additive, a zinc ionophore of hydroxychloroquine (HCQ) used in vivo treatment is being studied to inhibit the formation of Zn dendrites^23^. Additionally, hydrophilic functional groups were added to the PVDF surface by plasma treatment, which allowed for hydrogen bonding with water molecules and prevented side reactions brought on by H_2_O^24^. A surface-coating technique is used to provide an ultrathin layer of zinc vanadium oxide (ZVO) to a Zn anode surface. It has been demonstrated that this ZVO layer promotes homogeneous Zn deposition and prevents corrosion of the Zn anode^25^. Using vanadium (IV) oxide sulfate (VOSO_4_) as an electrolyte additive and a stable, high-performance active material as the anode/cathode to create high-energy performance ZIBs in a single step^26^. To get over these restrictions and improve the electrochemical behavior by making ZIBs safer and more wettable, a protective layer made of tellurium (Te) nanobelts has been added to the surface of the Zn anode^27^.

An increasing number of studies have indicated that alloying the zinc anode is a practical and efficient way to prevent HER without significantly raising the cost, which has shown a lot of promise for use. The effectiveness of a Sn alloying technique in preventing dendritic development and HER in a zinc metal electrode has been demonstrated^28–30^. Likewise, alloying with indium^31^, antimony^32^, and nickel^33^. It is commonly acknowledged that there are significant issues with global warming caused by CO_2_ gas emissions^34^. Fossil fuel combustion produces greenhouse gases, notably carbon dioxide. These emissions are recognized as the primary driver of severe climate change. Thus, significant global warming can be viewed as a challenging issue endangering human existence^35^. Because of this, some researchers attempted to use chemical conversion or physical capture to lower the atmospheric concentration of this gas^36,37^. As a result, Metal-CO_2_ battery systems present opportunities for developing high-performance energy storage solutions, thereby contributing to the advancement of electric vehicle infrastructure. These M-CO₂ systems are characterized by their ability to incorporate CO₂ gas directly as a chemical reactant. Because CO_2_ gas regulates the battery’s overall quantity, the battery showed a very high theoretical specific energy^38^. Sodium, lithium, aluminium, zinc, and potassium batteries were among the electrochemical storage devices of M-CO_2_ that used an attractive CO_2_ technique^39^. Therefore, CO_2_ batteries could use the metals mentioned above^40^. However, the literature review indicates that little research has been carried out regarding Zn or its alloyed form in the alkaline medium, which contains CO_2_^41^. Although a thorough examination of the literature showed several elements that were used as modifiers for an electrode made of zinc to slow down the process of electrode oxidation, bismuth was seldom studied as a zinc-based casting or doping element^14,42,43^. In addition, no prior studies have reported the fabrication of Zn–Bi alloys using a melt–fusing synthesis route for application in alkaline media. The present work introduces pioneering results on melt-synthesized Zn–Bi bimetallic anodes, which exhibit outstanding long-term stability, enhanced corrosion resistance, and superior operational performance. In this system, bismuth is incorporated at a trace level (0.5 wt%), serving as a strategic alloying element to mitigate corrosion processes and suppress hydrogen evolution in alkaline battery environments. This study systematically evaluates how this minor Bi addition influences the electrochemical durability, charge–discharge behavior, and overall lifetime of zinc-based anodes. The alloy’s corrosion behavior and electrochemical response were investigated in 1 M KOH solutions under both CO₂-free and CO₂-rich atmospheres, with pure zinc employed as a reference. A comprehensive methodological approach was adopted, such as galvanostatic cycling, electrochemical impedance spectroscopy (EIS), Tafel polarization, and advanced surface characterization using XRD, SEM, and EDX analyses to identify corrosion products and assess surface transformations. This integrated analysis provides a complete understanding of the performance of melt-processed Zn-Bi alloys in alkaline electrolytes and their potential for use in next-generation zinc-based energy storage systems.

## Experimental part

### Chemicals and materials

A 1 M KOH electrolyte was prepared by dissolving analytical-grade potassium hydroxide (British Drug Houses, BDH) in bidistilled water. CO₂-saturated electrolyte was obtained by continuously bubbling high-purity CO₂ gas through the 1 M KOH solution for approximately five hours at a controlled flow rate. The saturation process was monitored by measuring the solution pH, which stabilized at ~ 9, in contrast to the initial pH of ~ 13 for unsaturated KOH. High-purity zinc and bismuth metals (99.99%, Johnson Matthey) were used for alloy preparation. Working electrodes were fabricated in the form of circular disks with an exposed surface area of 0.5028 cm². Precise Zn/Bi metal mixtures were sealed in evacuated silica ampoules and subjected to melt-fusing at 750 °C for 24 h. The molten alloys were homogenized by intermittent agitation every three hours, followed by rapid quenching in an ice bath to ensure uniform solidification. Using this procedure, two electrode materials (pure zinc and zinc-0.5%bismuth) were successfully synthesized and employed as disk electrodes in the subsequent electrochemical investigations.

### Characterization

A Brucker AXS-D8 X-ray diffractometer was used to determine the crystalline phases of the manufactured electrodes at Cu-K_α_ (λ = 1.5418 Å). Electrode surface characterization employed SEM analysis. The JSM IT 200 microscope delivered detailed morphological data. An attached EDS detector enabled elemental mapping. Combined techniques analyzed working electrode surfaces comprehensively.

### Electrochemical investigation

Electrochemical testing employed a standard three-electrode setup. The VersaSTAT4 system provided potential and current control. Working electrodes were secured in Araldite mounts with Teflon masking. The active surface area measured precisely 0.5028 cm². Progressive polishing used decreasing grit sizes (800–1200). Acetone cleaning removed surface contaminants. Distilled water rinsing completed electrode preparation. Platinum functioned as the auxiliary electrode. Saturated Ag/AgCl provided the reference potential. Pre-treatment involved cathodic polarization at -2 V. Five-minute cleaning removed surface oxides. Bubble removal preceded analytical measurements.

#### Tafel technique

At different temperatures (25–50 °C) and voltage ranges (-0.25 V and + 0.25 V), Tafel diagrams were produced compared to the open-circuit voltage steady state (*E*_corr_.) with a rate of scan about 1 mVs^− 1^.

#### EIS technique

In the EIS tests, frequencies ranging from 10^4^ Hz to 1 Hz, direct DC voltages of ± 100 mV, and 10 mV AC voltage were used.

#### Charge-discharge technique

With a voltage limitation of 0 volts, the charge-discharge measurements were carried out using unchanged charge-discharge currents of ± 2, ±4, ± 6, and ± 8 mA cm^− 2^.

### Assessing the corrosion parameters

For half an hour, the working electrodes were left to settle at the E_corr_, in an electrolyte of 1 M KOH. The corrosion current densities (*i*_corr_) are calculated using the extrapolated crossing point of the anodic and cathodic Tafel lines for each electrode under investigation, which were carefully determined. Cleaned electrodes and freshly made solution were utilized in every investigation. The temperatures used for the trials were 25, 35, 45, and 50 degrees Celsius using a CW3-10P heating bath circulator, which has powerful pumping capabilities and both internal and external circulation. The identical tests were performed at least three times with excellent reproducibility. All the investigations described earlier have been repeated.

##  Results and discussion: the behaviour of Zn and Zn-Bi anodes in KOH with and without CO_2_

### Tafel polarization

**Fig. 1:**
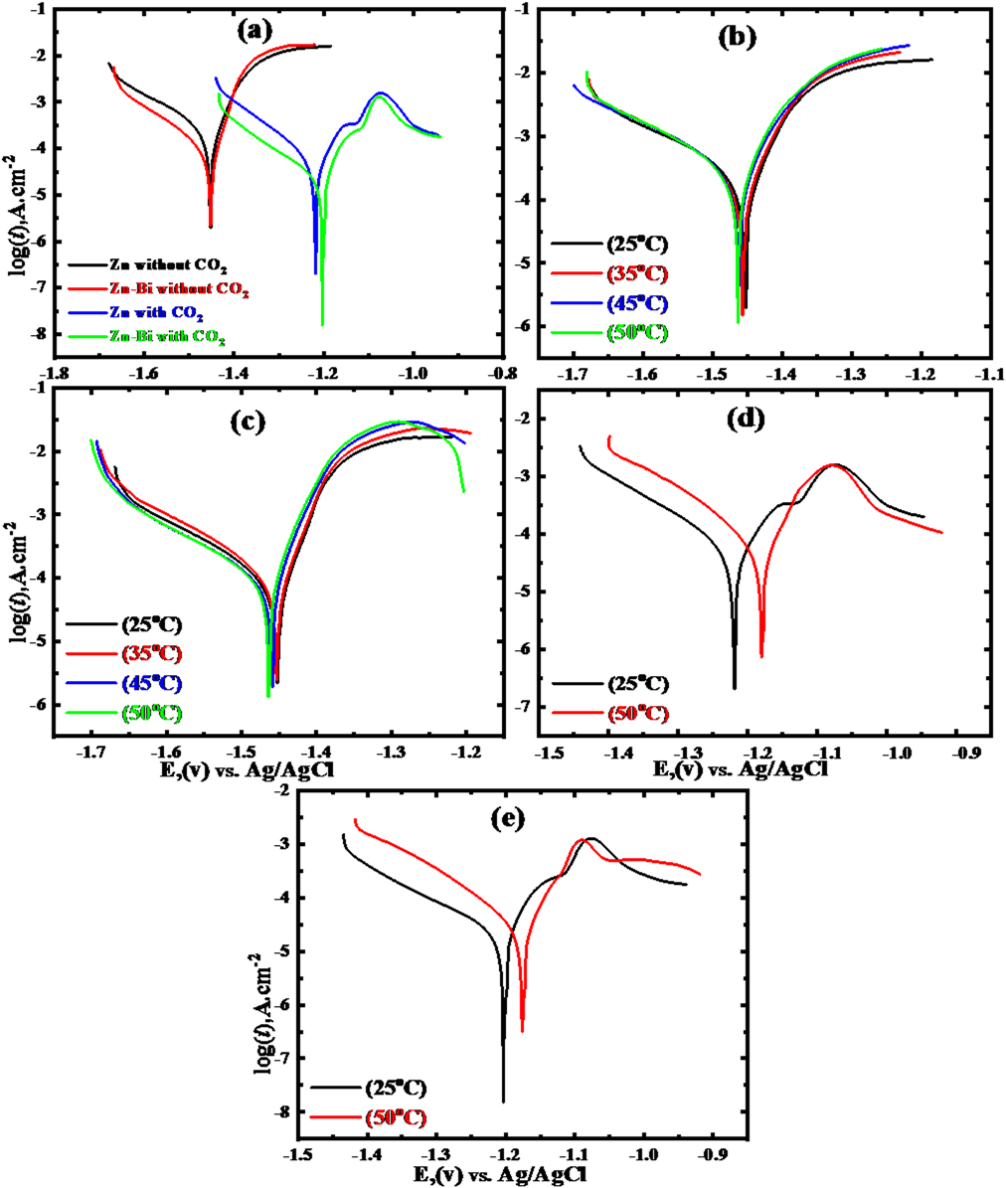
Tafel polarization Plots of Zn and its alloy in KOH at 25 °C without and with CO_2_ (**a**), (**b,c**) at various temperatures (25–50 °C) in the absence of CO_2_, (**d,e**) at (25, 50 °C) in the presence of it and 1 mVs^-1^ scan rate, respectively.

Tafel polarization plots for pure Zn and Zn-Bi in the alkaline media at ambient temperature (25 °C), excluding and including dissolved CO_2_, are displayed in Fig. 1a. The findings revealed that the Zn-Bi electrode consistently exhibited reduced cathodic and anodic branch current densities compared to the pure Zn electrode in 1 M KOH, under both CO_2_-free and CO_2_-containing conditions. This behavior stems from bismuth’s capacity to retard zinc corrosion processes. Unlike pure zinc, bismuth functions as an alloying component with notable hydrogen evolution characteristics and contributes to corrosion rate reduction in zinc alloy through Bi_2_O_3_ formation. Nevertheless, relative to pure zinc, the experimental data showed that anodic and cathodic current density curves are diminished in the zinc-0.5% Bi alloy. This implies that zinc alloying with 0.5% Bi enhanced the hydrogen overpotential of zinc^19^. CO₂ saturation of KOH solution generates dual beneficial effects on electrode corrosion. Current density values drop significantly while potential measurements move in the positive direction, confirming improved corrosion performance as shown in Table 1. This pattern can be attributed to the reaction of the KOH electrolyte and CO_2_ gas to produce K_2_CO_3_, which then combines with zinc ions to produce insoluble ZnCO_3_ on the surface. In contrast to its absence, the formation of Zn(OH)_2_ results in a greater corrosion current. The development of a non-adhered, porous layer on the surface is responsible for this behaviour^31^.

In contrast to its absence, the anodic branch of Zn and Zn-0.5%Bi exhibits a knee in the presence of CO_2_. As a result, this pattern can be explained by the surface development of insoluble ZnCO_3_ and its hydroxide, which will be discussed in more detail using XRD data. Note that under these conditions, CO_2_ dramatically lowers the negative potential, current, and corrosion rate for two electrodes. According to previous studies, carbon dioxide raises corrosion current and, consequently, the rate of corrosion. These findings contradict these results. The development of an adsorbent protective layer on the electrodes being studied explains this. Additionally, CO_2_ reacts with the KOH solution to form K_2_CO_3_ and/or KHCO_3_, which can react with zinc ions to form ZnCO_3_. This causes the electrode surface to become coated with the insoluble metal carbonate. When CO_2_ is present, the developed protective layer is composed of metal carbonate. Within the Tafel potential range, the anodic and cathodic voltages vs. the density of current were analysed to estimate the corrosion parameters^44^ as shown in Table 1.

The determination of *i*_corr_ employed Tafel line extrapolation to corrosion potentials for all tested materials. CO₂ presence produces marked shifts in polarization behavior, driving both reaction branches toward lower current densities. Zinc electrodes show remarkable improvement, with corrosion rates falling from 99.311 µA.cm⁻² in pure KOH to 26.92 µA.cm⁻² when CO₂ is introduced. Zn-Bi alloys achieve superior performance, reducing from 41.399 µA.cm⁻² to an exceptional 12.3 µA.cm⁻² under CO₂ conditions at 25 °C. These pronounced decreases establish CO₂ as a highly effective corrosion inhibitor for zinc-based materials in alkaline media. The inhibition operates through mixed-type mechanisms, simultaneously suppressing anodic metal dissolution and cathodic hydrogen generation.

The following formula can be used to determine the inhibitory efficiency (η%):

1 $$\:{\upeta\:}\%=\frac{{i}_{p}-{i}_{w}}{{i}_{p}}\times\:100\:\:\:\:\:\:\:\:\:\:\:$$

where *i*_p_ and *i*_w_ refer to the density of corrosion current in 1 M KOH without carbon dioxide and in a solution with it, respectively. The presence of CO_2_ is observed to raise the η%. Furthermore, the results demonstrated that in all cases, the alloy’s η% is greater than the metal’s comparable value. This suggests a greater amount of the metal’s oxide is generated and adheres to the surface, which results in the surface’s protective effect. Bismuth is a small alloying element that minimizes the quantity of active sites in relation to surface area. This leads to the layer of oxide that forms being more adsorbable. These data suggest that covering the anodic and cathodic areas can delay electrode processes.

The E_corr_. for the Zn electrode is the same as its alloy in pure medium, but there is a positive shift when CO_2_ is added to the solution, as shown in Table 1. The following formula can be used to determine the average corrosion rate (U_corr_.), which correlates with the *i*_corr_.

2 $$\:{U}_{corr.}\left(\frac{mm}{y}\right)=\frac{3270\times\:M\times\:{i}_{corr.}}{d}\:\:\:\:\:\:\:\:\:\:\:\:\:\:\:\:\:$$

The unit of U_corr_. is determined by the constant 3270, M stands for equivalent weight (g.equiv.^−1^), d for the corroding material density (g/cm^3^), and *i*_corr_. for the density of corrosion current (A/cm^2^). Calculating the U_corr_. for both tested electrodes revealed a decrease with the presence of carbon dioxide gas in the electrolyte and alloying.

Figure 1a Tafel polarization diagram demonstrating that cathodic and anodic current density branches substantially diminish when CO_2_ is introduced compared to its absence. The *i*_corr_ densities reduce from 99.311 to 41.399 to 26.92 and 12.3 µA.cm^− 2^ for Zn and Zn-Bi electrodes, respectively. Additionally, the *E*_corr_ measurements for zinc and its alloy move from a more cathodic direction (-1.45 V) without CO_2_ to a more anodic direction (-1.2 V) with CO_2_ present. Moreover, the η% of Zn and Zn-Bi electrodes rises substantially, achieving 72.893% and 87.615%, respectively, under CO_2_ conditions. The *i*corr and Ucorr of the investigated electrodes reduce considerably when the alkaline environment includes CO_2_^45^. The examined data contradict the already published findings in neutral and acidic solutions with CO_2_. A higher *i*_corr_ and, thus, a higher U_corr_ are caused by the presence of CO_2_^46^. The electrochemical efficiency of alkaline batteries may therefore be new as a result of the current findings.

Potentiodynamic curves of polarization are used to calculate the Resistance of Polarization (R_P_), which in turn determines the porosity (P_R_) of Zn alloy^47^.

3 $$\:{R}_{p}=\frac{\beta\:}{{i}_{corr.}}\:,\:\:\:\:\:\:\beta\:=\frac{{b}_{a\:}{b}_{c}}{2.303\left({b}_{a}+{b}_{c}\right)}\:\:\:\:\:\:\:$$

While

4 $$\:{P}_{R}\left(\%\right)=\frac{{R}_{P}^{^\circ\:}}{{R}_{P}}\times\:100$$

where R^o^_p_ is pure zinc’s polarization resistance, R_p_ is the Zn alloy’s polarization resistance, and P_R_ is the total porosity.

In comparison to pure zinc (100%), the porosity percent (P_R_%) of Zn-Bi alloy (67.078%) is lower. It has been noted that the addition of bismuth to zinc caused a significant drop in porosity percentage. Consequently, as compared to zinc, the Zn-Bi alloy exhibits greater corrosion resistance. Thus, the information gleaned from impedance tests and Tafel plots supports this pattern.

In the present study, the HER behavior of pure Zn and Zn-0.5 wt% Bi electrodes was evaluated through cathodic polarization and Tafel analysis under both CO₂-free and CO₂-saturated conditions.

The results indicate that bismuth alloying significantly suppresses HER activity, as evidenced by lower cathodic current densities and increased HER overpotentials compared to pure zinc. This behavior can be attributed to the modification of the zinc surface electronic structure by bismuth, which reduces the availability of active sites for hydrogen adsorption and slows down the HER kinetics. The observed increase in Tafel slopes further suggests a change in the rate-determining step of the hydrogen evolution process.

The introduction of CO₂ into the alkaline electrolyte provides an additional suppression mechanism. CO₂ exposure promotes the in situ formation of a ZnCO₃-based surface layer, which acts as a physical and chemical barrier that limits proton access and hinders hydrogen bubble nucleation. As a result, HER is effectively mitigated, leading to reduced parasitic reactions and enhanced electrode stability. This synergistic effect of bismuth alloying and CO₂-induced surface passivation explains the improved electrochemical performance and prolonged charge-discharge stability observed for the Zn-Bi anodes.

From Table 1, the cathodic Tafel slopes (βc) obtained for Zn and Zn-Bi electrodes in alkaline media range between ~ 75 and 120 mV·dec⁻¹, indicating that the hydrogen evolution reaction proceeds predominantly via a Volmer–Heyrovsky mechanism. The relatively high βc values suggest that the Volmer step (water discharge) is the rate-determining step, particularly for the Zn-Bi alloy. The presence of CO₂ does not alter the fundamental HER mechanism but significantly suppresses HER kinetics by promoting the formation of a ZnCO₃-based protective surface layer, which limits hydrogen adsorption and bubble nucleation.

#### Effect of temperature

Figure 1b, c shows the behaviour of cathodic and anodic polarization of Tafel lines for Zn and Zn-Bi in 1 M KOH without carbon dioxide gas, respectively, where the temperature influence ranged from 25 to 50 °C at a 1 mVs^− 1^ scan rate. According to the cathodic and anodic lines’ slopes on the Tafel polarization plots, the corrosion parameters were precisely calculated. *i*_corr_ values for zinc and Zn-Bi systems were also obtained through standard Tafel extrapolation methodology to their respective *E*_corr_ points. The overall morphology of electrochemical polarization curves remains essentially constant across different temperature conditions for both anodic and cathodic processes. This demonstrates the stability of the zinc electrode and its alloy at high temperatures. It is important to note that the lines slightly shift to the upside as the electrolyte temperature rises for Zn and Zn-Bi alloy. This behaviour aligned with the findings of El-Sayed et al.^32^. Rising temperature conditions drive both cathodic and anodic Tafel branches toward increased current density values for the alloy system when compared to pure zinc electrode behavior. The polarization curves show measurable but moderate current enhancement with temperature elevation across both reaction processes. This could mean that, concerning the Zn-Bi alloy, the rate of anodic dissolution and hydrogen gas evolution appears to be slightly accelerated with increasing temperature. The presence of small amounts of Bi alloyed with zinc can explain this phenomenon. As a result, there is an increase in the over-potential of hydrogen and a delay in anodic dissolution on the alloy surface. In addition, the alloy surface’s hydrogen evolution process requires a significant overvoltage within the temperature range under study^31^. Thus, the substantial improvement in corrosion resistance for the alloy over pure zinc can be ascribed to this tendency. Table 1 lists the corrosion characteristics and protection efficiency (η%) for Zn and Zn-Bi at various temperature ranges. As the temperature increases, the *E*_corr_. The value for zinc and its alloy shifts slightly towards a more negative potential and appears to be similar over the temperature ranges. Accordingly, it seems that temperature has roughly the same impact on cathodic and anodic processes^33^. The corrosion protection efficiency (Table 1) progressively drops as the temperature rises, reaching its lowest value at 50 °C (53.118%). This indicates that compared to pure zinc, the alloy under research has better resistance to corrosion, especially at lower temperatures. This can be ascribed to the zinc atom is smaller than the bismuth atom, the alloy’s active site density is reduced. Because the bismuth atoms shield the zinc surface, this inhibits the anodic breakdown of zinc and the evolution of hydrogen.

Data in Table 1 reveal that temperature increases drive higher *i*_corr_ values for both samples when CO₂ gas is introduced to KOH solutions, Fig. 1d, e. The reduction in protection efficiency (η%) at elevated temperatures stems from thermal degradation of the protective surface films on the electrodes.^48^. For the two electrodes under study, this behaviour is explained by a little drop in ZnCO_3_ levels on the electrode surface. This suggests that temperature elevation to 50 °C disrupts the normal formation processes of ZnCO₃ and ZnO compounds that occur on Zn and Zn-Bi alloy surfaces at 25 °C.

Consequently, the electrochemical reactions that may take place on the surface of Zn or Zn-Bi in KOH electrolyte (without CO_2_) at 25 °C is possible to demonstrate below:

For pure zinc

5 $$\:6\mathrm{Z}\mathrm{n}+12\mathrm{O}{\mathrm{H}}^{-} \rightarrow \:6\mathrm{Z}\mathrm{n}{\left(\mathrm{O}\mathrm{H}\right)}_{2}+12{\mathrm{e}}^{-}\:\:\:\:\:\:\:\:\:\:\:\:\:\:\:\:\:\:\:\:\:\:\:\:\:\:\:\:\:\:\:\:\:\:\:\:\:\:\:\:\:\:\:\:\:\:\:\:\:\:\:\:\:\:\:\:\:\:\:\:\:\:\:\:\:\:\:\:\:\:\:\:\:\:\:\:\:\:\:\:\:\:\:\:\:\:\:\:\:\:\:\:\:\:\:\:\:\:\:\:\:\:$$

For its alloy

6 $$\:\mathrm{Z}\mathrm{n}+2\mathrm{O}{\mathrm{H}}^{-} \rightarrow \:\mathrm{Z}\mathrm{n}\mathrm{O}+{\mathrm{H}}_{2}\mathrm{O}+2{\mathrm{e}}^{-}\:\:\:\:\:\:\:\:\:\:\:\:\:\:\:\:\:\:\:\:\:\:\:\:\:\:\:\:\:\:\:\:\:\:\:\:\:\:\:\:\:\:\:\:\:\:\:\:\:\:\:\:\:\:\:\:\:\:\:\:\:\:\:\:\:\:\:\:\:\:\:\:\:\:\:\:\:\:\:\:\:\:\:\:\:\:\:\:\:\:\:\:\:\:\:\:\:\:\:\:\:\:\:\:\:\:\:\:$$

7 $$\:2\mathrm{B}\mathrm{i}+6\mathrm{O}{\mathrm{H}}^{-} \rightarrow \:{\mathrm{B}\mathrm{i}}_{2}{\mathrm{O}}_{3}+{3\mathrm{H}}_{2}\mathrm{O}+6{\mathrm{e}}^{-}\:\:\:\:\:\:\:\:\:\:\:\:\:\:\:\:\:\:\:\:\:\:\:\:\:\:\:\:\:\:\:\:\:\:\:\:\:\:\:\:\:\:\:\:\:\:\:\:\:\:\:\:\:\:\:\:\:\:\:\:\:\:\:\:\:\:\:\:\:\:\:\:\:\:\:\:\:\:\:\:\:\:\:\:\:\:\:\:\:\:\:\:\:\:\:\:\:\:\:\:$$

However, the following reaction mechanism can be anticipated for the two electrodes under investigation in KOH containing CO_2_ gas:

8 $$\:6\mathrm{Z}\mathrm{n}+12\mathrm{O}{\mathrm{H}}^{-} \rightarrow \:\:6\mathrm{Z}\mathrm{n}{\left(\mathrm{O}\mathrm{H}\right)}_{2}+12{\mathrm{e}}^{-}\:\:\:\:\:\:\:\:\:\:\:\:\:\:\:\:\:\:\:\:\:\:\:\:\:\:\:\:\:\:\:\:\:\:\:\:\:\:\:\:\:\:\:\:\:\:\:\:\:\:\:\:\:\:\:\:\:\:\:\:\:\:\:\:\:\:\:\:\:\:\:\:\:\:\:\:\:\:\:\:\:\:\:\:\:\:\:\:\:\:\:\:\:\:\:\:\:\:\:\:\:$$

9 $$\:3\mathrm{C}{\mathrm{O}}_{2}+6\mathrm{K}\mathrm{O}\mathrm{H} \rightarrow \:\:{3\mathrm{K}}_{2}\mathrm{C}{\mathrm{O}}_{3}+{3\mathrm{H}}_{2}\mathrm{O}\:\:\:\:\:\:\:\:\:\:\:\:\:\:\:\:\:\:\:\:\:\:\:\:\:\:\:\:\:\:\:\:\:\:\:\:\:\:\:\:\:\:\:\:\:\:\:\:\:\:\:\:\:\:\:\:\:\:\:\:\:\:\:\:\:\:\:\:\:\:\:\:\:\:\:\:\:\:\:\:\:\:\:\:\:\:\:\:\:\:\:\:\:\:\:\:\:\:\:\:\:\:\:\:$$

10 $$\:6\mathrm{Z}\mathrm{n}{\left(\mathrm{O}\mathrm{H}\right)}_{2}+{3\mathrm{K}}_{2}\mathrm{C}{\mathrm{O}}_{3} \rightarrow \:\:\mathrm{Z}\mathrm{n}\mathrm{C}{\mathrm{O}}_{3}+{\mathrm{Z}\mathrm{n}}_{5}\left(\mathrm{O}\mathrm{H}{)}_{6}\right(\mathrm{C}{\mathrm{O}}_{3}{)}_{2}+6\mathrm{K}\mathrm{O}\mathrm{H}\:\:\:\:\:\:\:\:\:\:\:\:\:\:\:\:\:\:\:\:\:\:\:\:\:\:\:\:\:\:\:\:\:\:\:\:\:\:\:\:\:\:\:\:\:\:\:\:$$

The above equations show how zinc and a KOH solution with CO_2_ gas partially react at 25 °C. The overall response, however, can be expressed as follows:

11 $$\:6\mathrm{Z}\mathrm{n}+12\mathrm{O}{\mathrm{H}}^{-}+3\mathrm{C}{\mathrm{O}}_{2} \rightarrow \:\:\mathrm{Z}\mathrm{n}\mathrm{C}{\mathrm{O}}_{3}+{\mathrm{Z}\mathrm{n}}_{5}\left(\mathrm{O}\mathrm{H}{)}_{6}\right(\mathrm{C}{\mathrm{O}}_{3}{)}_{2}+{3\mathrm{H}}_{2}\mathrm{O}+12{\mathrm{e}}^{-}\:\:\:\:\:\:\:\:\:\:\:\:\:\:\:\:\:\:\:\:\:\:\:\:\:\:\:\:\:\:\:$$

As a result, the components (ZnCO_3_ and Zn_5_(OH)_6_(CO_3_)_2_) that were derived from XRD concur with those proposed by Eq. (11).

Corrosion activation energies for zinc and Zn-Bi electrode systems were determined through Arrhenius equation analysis, as shown below:

12 $$\:\mathrm{ln}{i}_{\mathrm{c}\mathrm{o}\mathrm{r}\mathrm{r}.\:}=\frac{-{\mathrm{E}}_{\mathrm{a}}}{\mathrm{R}\mathrm{T}}+\mathrm{ln}A\:\:\:\:\:\:\:\:\:\:\:\:\:\:\:\:\:\:\:\:\:\:\:\:\:\:\:\:\:\:\:\:\:\:\:\:\:\:\:\:\:\:\:\:\:\:\:\:\:\:\:\:\:\:\:\:\:\:\:\:\:\:\:\:\:\:\:\:\:\:\:\:\:\:\:\:\:\:\:\:\:\:\:\:\:\:\:\:\:\:\:\:\:\:\:\:\:\:\:\:\:\:\:\:\:\:\:\:\:\:\:\:\:\:\:\:\:\:\:\:\:\:\:\:\:\:\:\:\:\:\:\:$$

**Fig. 2:**
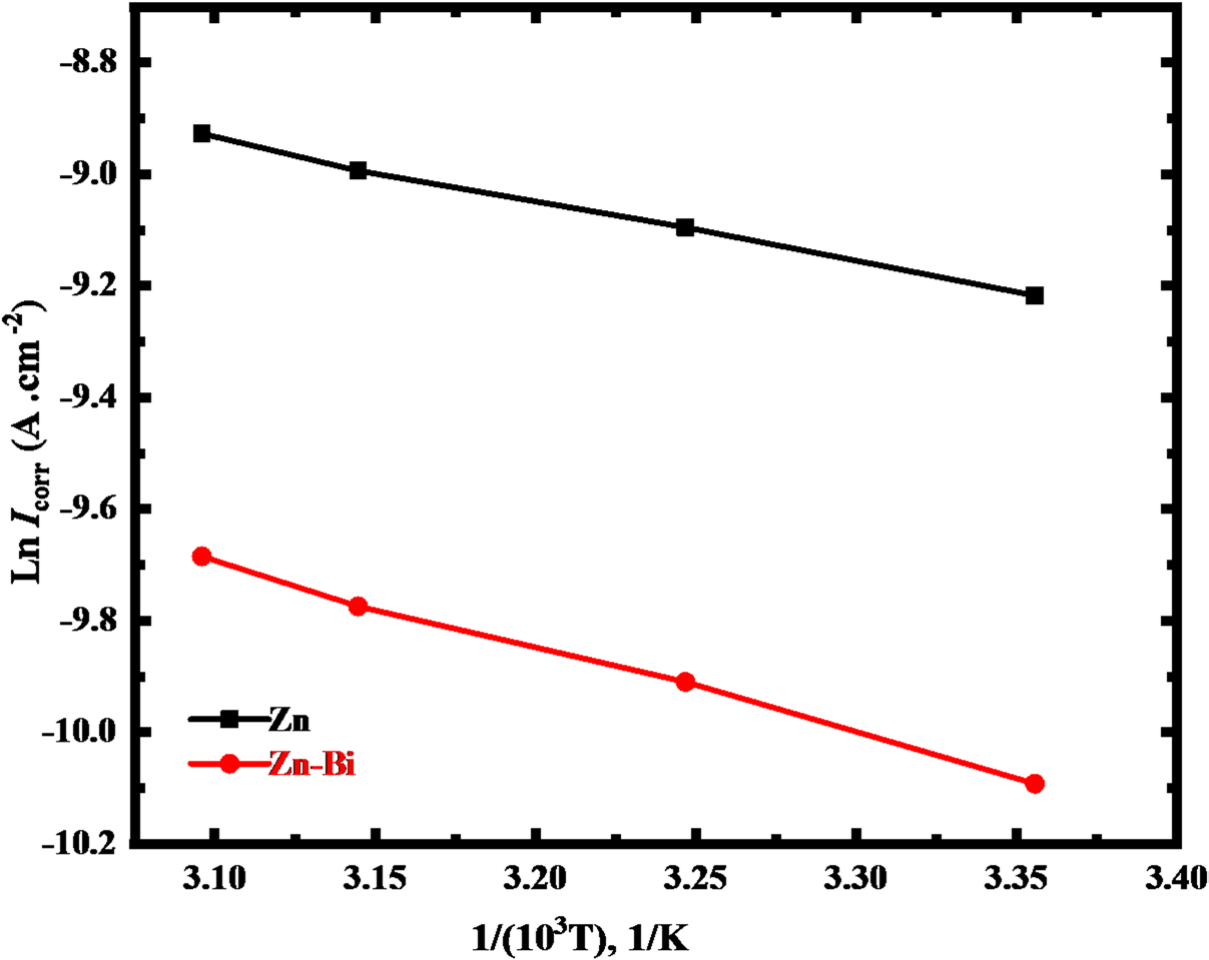
Plots of Arrhenius from 25 to 50 °C for Zn and Zn-Bi in KOH.

E_a_ is the measured energy of activation in kJ mol^− 1^, the universal constant of gases in J mol^−1^K^− 1^ is R, A is the pre-exponential constant, and the absolute temperature in Kelvin is T. As indicated in Fig. 2, the slope of ln *i*_corr_. vs. 1/T plotted lines were used to precisely determine the E_a_ values of Zn-Bi alloy and pristine Zn anodes. Experimental findings show increased E_a_ values for Zn-Bi electrodes versus pure zinc in 1 M KOH electrolyte (see Table 2). The enhanced activation energy barrier correlates with bimetallic phase formation in the solid solution, effectively impeding electrochemical corrosion reaction kinetics. Consequently, the electrochemical corrosion rate of the synthesized alloy was decreased^31^. The limited number of active sites in the alloy may be the cause of the increase in E_a_ values; thus, the cathodic to anodic area ratio increased. El-Sayed et al.^49^ demonstrated that the hydrogen evolution reaction was reduced or inhibited when a solid solution contains only one phase as a result of alloying. It is confirmed that the optimum protection at the slowest rate of corrosion is provided by the homogeneity of this phase in the produced alloy. This attitude toward the rate of zinc and Zn-Bi electrodes’ electrochemical corrosion was validated by the computed E_a_.

**Table 1:** Corrosion parameters of Tafel polarization for Zn and its alloy were determined in KOH without and with CO_2_ at different temperatures.

Electrolytes	Electrodes	Parameters					
		i_corr_. (µA / cm^2^)	- E_corr_. (V vs. Ag/AgCl)	β_a_(mV/ decade)	- β_c_ (mV/ decade)	U_corr._ (mm/y)	η%
**25 °C**							
KOH	Zn	99.311	1.452	43.94	85.09	1.487	-
	Zn-Bi	41.399	1.452	32.14	83.24	0.620	58.313
KOH + CO_2_	Zn	26.92	1.217	51.68	75.03	0.403	72.893
	Zn-Bi	12.3	1.203	37.92	87.74	0.184	87.615
**35 °C**							
KOH	Zn	112.201	1.456	45.78	90.38	1.680	-
	Zn-Bi	49.69	1.453	32.90	85.63	0.744	55.713
**45 °C**							
KOH	Zn	124.165	1.462	47.18	80.98	1.859	-
	Zn-Bi	56.885	1.460	34.75	107.14	0.852	54.186
**50 °C**							
KOH	Zn	132.739	1.463	41.78	82.64	1.988	-
	Zn-Bi	62.230	1.464	36.62	119.79	0.933	53.118
KOH+CO_2_	Zn	39.37	1.179	41.85	82.38	0.589	65.58
	Zn-Bi	18.197	1.176	39.69	81.77	0.2728	83.99

**Table 2:** The electrochemical corrosion reaction’s E_a_ values for Zn and Zn-Bi in 1 M KOH.

Electrode	E_a_ (KJ/mole)
Zn	9.114
Zn-Bi	12.776

### Characterization of the corroded surfaces of Zn and its alloy in KOH

**Fig. 3:**
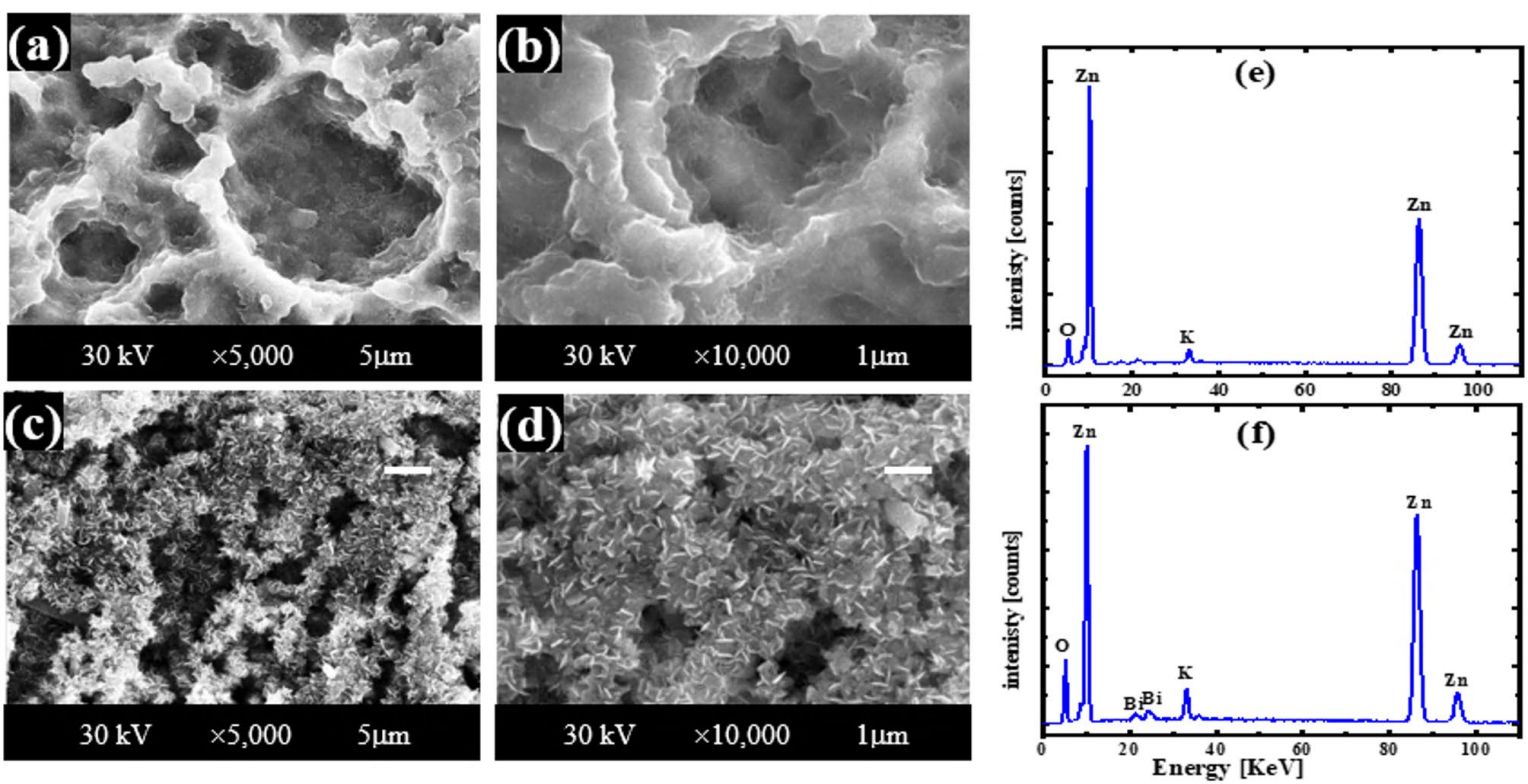
SEM images taken at a magnification of 5,000 and 10,000, and EDX analysis charts of the corroded layer of zinc (**a,b,e**) and its alloy (**c,d,f**) at 25 °C in pure 1 M KOH at the active region, respectively.

**Fig. 4:**
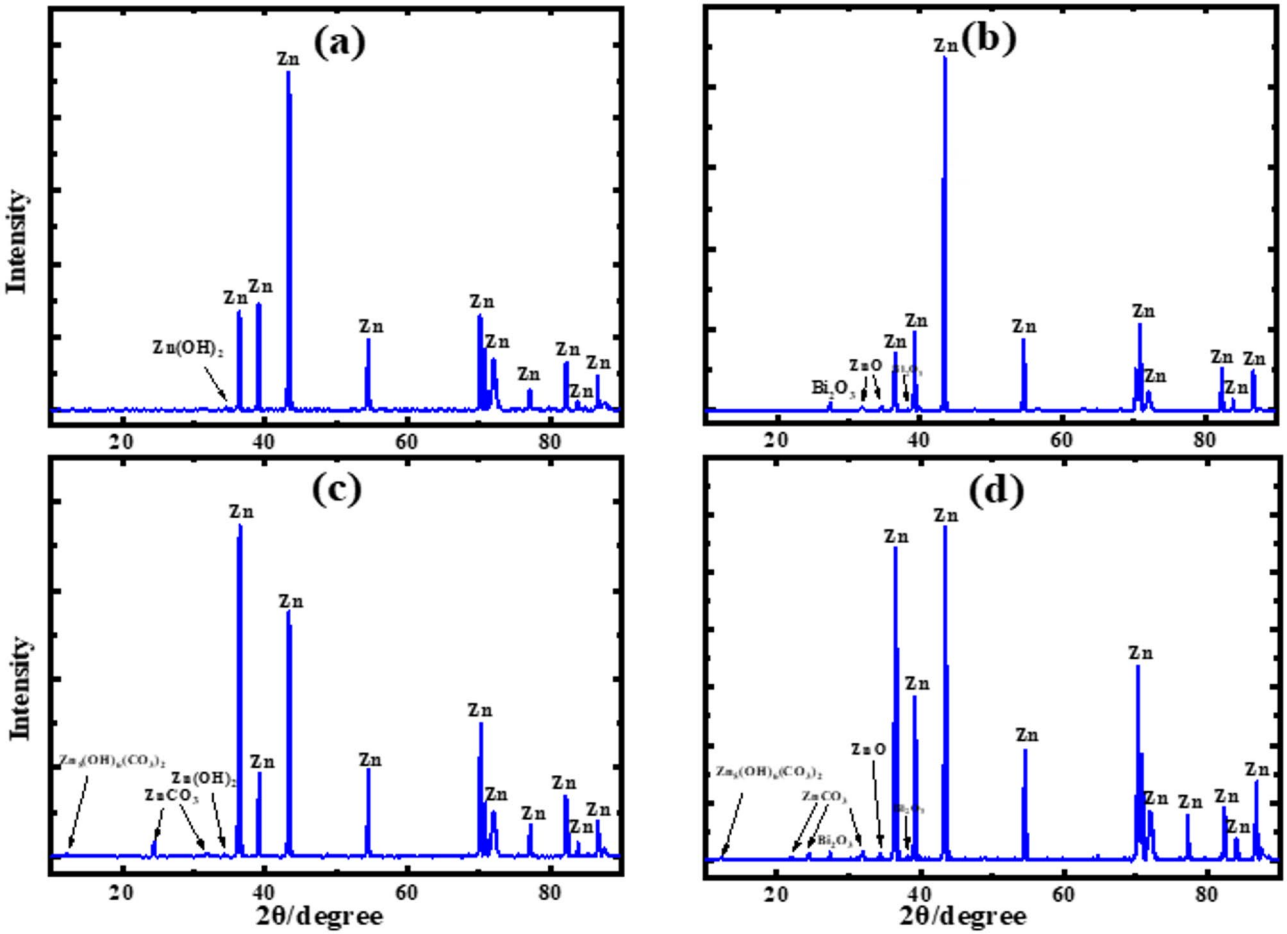
XRD electrochemical products generated on the zinc surfaces (**a,c**) and Zn-Bi (**b,d**), at 25 °C in KOH without and with CO_2_ in the region of activity, respectively.

Figure 3 displays SEM images and EDX spectra of corrosion products formed on pure zinc and Zn-Bi electrodes after exposure to 1 M KOH at 25 °C, captured at ×5,000 and ×10,000 magnifications within the active potential region. Pure zinc surfaces develop thick, porous corrosion layers with poor adhesion that cover nearly the complete electrode area, as shown in Fig. 3 (a, b). These loosely-bound particles exhibit varied morphologies characteristic of Zn(OH)₂ formation, confirmed by post-corrosion XRD analysis in Fig. 4 (a). The Zn-Bi alloy surface presents markedly different behavior, with Fig. 3 (c, d) revealing smaller, more tightly-bonded particles that adhere strongly to the substrate. Bismuth addition as a minor alloying element effectively suppresses Zn(OH)₂ formation, instead promoting ZnO and Bi₂O₃ development as verified by XRD results in Fig. 4 (b). EDX analysis in Fig. 3 (e, f) and data Table 3 quantifies elemental composition for both materials in CO₂-free KOH electrolyte, supporting the observed lower *i*_corr_. values for the alloy. The enhanced corrosion resistance results from protective oxide film formation, where Bi₂O₃ and ZnO create superior barrier properties compared to the hydroxide layer on pure zinc.

**Fig. 5:**
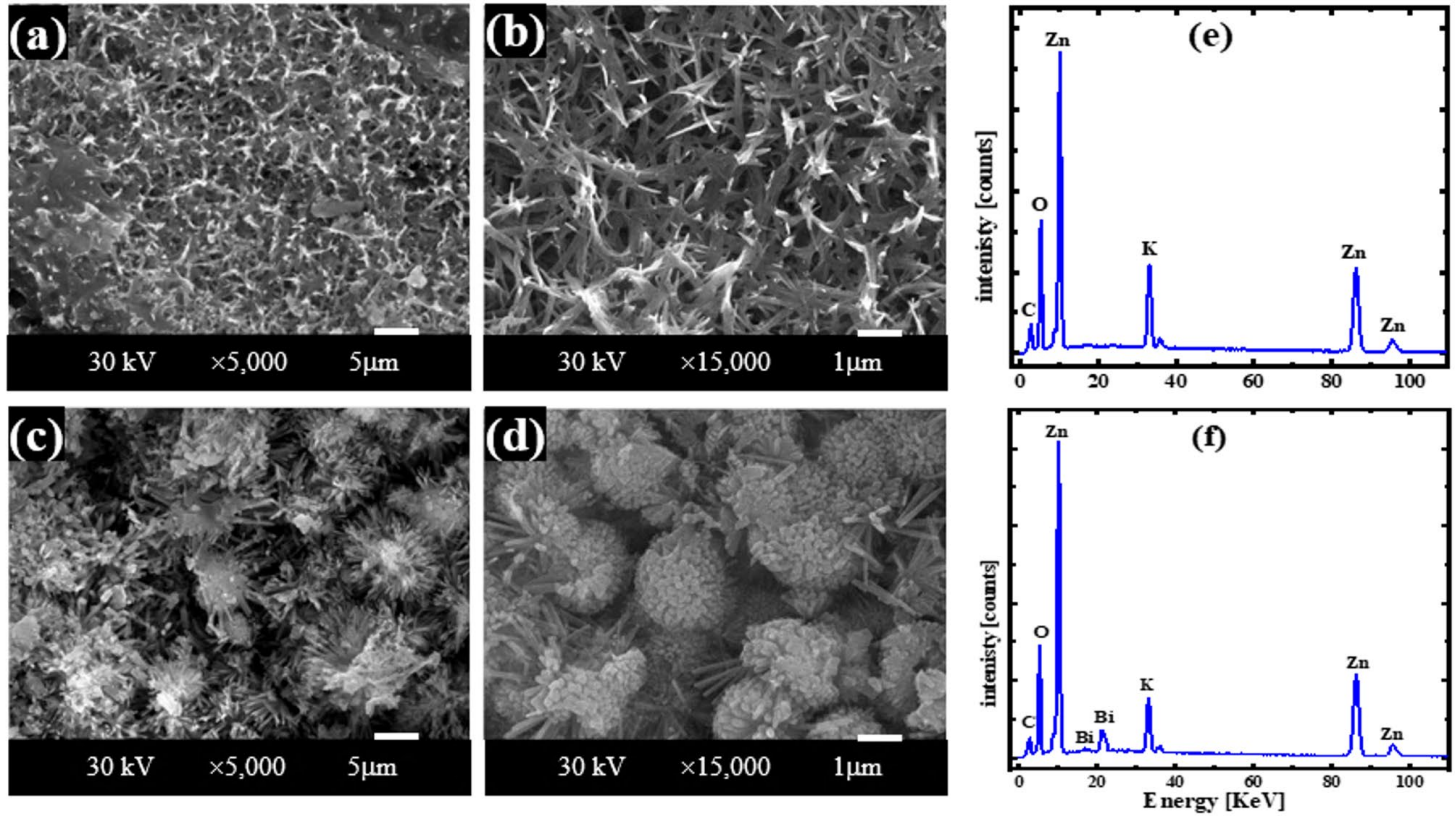
SEM images taken at a magnification of 5,000 and 15,000, and EDX analysis charts of the corroded layer of zinc (**a,b,e**) and its alloy (**c,d,f**) at 25 °C in KOH with CO_2_ at the active region, respectively.

Figure 5 The SEM pictures and EDX spectrum of the resulting corrosion products at the zinc and Zn-Bi electrodes in KOH electrolyte with CO_2_ at 25 °C within the region of activity, enlarged by ×5,000 and ×15,000, respectively. The surface of pure zinc is nearly entirely coated in a thick film of corrosion substance that is weakly adhered to it and is porous, as seen in Fig. 5 (a, b). The different morphologies of the particles in this layer may be connected to the production of the compounds Zn(OH)_2_ and ZnCO_3_. The newly generated phase of ZnCO_3_ was apparent on the electrode surface in the data of XRD for Zn and Zn-Bi in 1 M KOH saturated with carbon dioxide, as illustrated in Fig. 4 (c, d), respectively, in contrast to the absence of CO_2_. This suggests that the saturated KOH solution with CO_2_ may delay the surface synthesis of Zn(OH)_2_ or ZnO, which takes place in pure KOH solution. This implies that the surface generation of insoluble ZnCO_3_ when CO_2_ is present inhibits the corrosion process. When Zn(OH)_2_ or ZnO synthesis delays and ZnCO_3_ is generated on the electrode surface, its dissolution is significantly decreased. However, the alloy’s image indicates that compared to the zinc surface, the particles are smaller and more tightly attached. Therefore, the synthesis of Zn(OH)_2_ is delayed when bismuth is present as a tiny alloying component with zinc. In addition to ZnCO_3_, these particles are believed to be the product of the combination of ZnO and Bi_2_O_3_. Figure 5 (e, f) shows the EDX analysis graphs and data Table 4 for Zn and Zn-0.5%Bi in KOH with CO_2_, respectively. This result confirms that the alloy has a lower *i*_corr_ than pure zinc. SEM images show that more protection is offered by zinc in a solid solution containing bismuth, because of the production of its oxide layer (Bi_2_O_3_). In addition, the corrosion current density for two specimens is lower when carbon dioxide is present than in its absence because of the formation of zinc carbonate. This alloy is therefore seen to be a promising substance for alkaline batteries with a long lifespan.

The obtained XRD patterns were indexed using standard reference data, including hexagonal Zn (ICDD/JCPDS No. 00-004-0831), rhombohedral Bi (ICDD/JCPDS No. 01-085-1331), rhombohedral ZnCO₃ (ICDD/JCPDS No. 00-008-0449), and monoclinic Zn(OH)₆(CO₃)₂ (ICDD/JCPDS No. 00-014-0256).

**Table 3:** EDX data of the layer of oxide that forms on the surfaces of Zn and Zn-Bi in KOH in the active region.

Anode	Element	Mass%	Atom%
Zn	O	13.18 ± 0.19	37.84 ± 0.56
	K	2.43 ± 0.06	2.85 ± 0.07
	Zn	84.39 ± 0.43	59.31 ± 0.30
	Total	100.00	100.00
Zn-Bi	O	20.01 ± 0.24	50.69 ± 0.61
	K	3.32 ± 0.06	3.44 ± 0.07
	Zn	72.75 ± 0.39	45.11 ± 0.24
	Bi	3.92 ± 0.15	0.76 ± 0.03
	Total	100.00	100.00

**Table 4:** EDX data of the layer of oxide that forms on the surfaces of Zn and Zn-Bi in KOH with CO_2_ in the active region.

Electrode	Element	Mass%	Atom%
Zn	C	10.64 ± 0.10	24.98 ± 0.25
	O	25.34 ± 0.17	44.67 ± 0.31
	K	9.43 ± 0.09	6.80 ± 0.06
	Zn	54.59 ± 0.40	23.55 ± 0.17
	Total	100.00	100.00
Zn-Bi	C	8.13 ± 0.10	21.07 ± 0.26
	O	22.68 ± 0.17	44.15 ± 0.33
	K	7.11 ± 0.08	5.66 ± 0.06
	Zn	60.64 ± 0.44	28.90 ± 0.21
	Bi	1.44 ± 0.10	0.22 ± 0.02
	Total	100.00	100.00

### EIS measurements

**Fig. 6:**
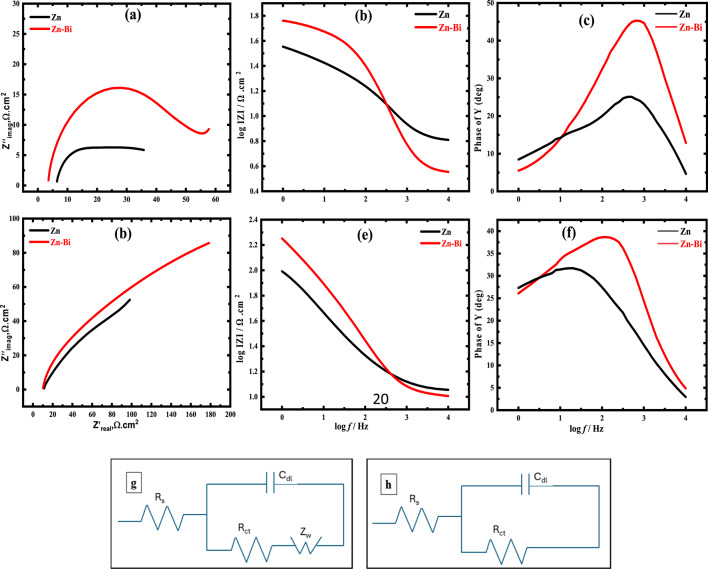
Nyquist diagrams (**a,d**), Bode magnitude plots (**b,e**), and phase angle responses (**c,f**) of Zn and its alloy in KOH without and with CO₂ exposure, respectively, along with analogous circuit models (**g,h**). Measurements were made with the circuit potential open using a 10 mV AC amplitude at 25 °C across a 10^4^ Hz to 1 Hz frequency range.

EIS results for Zn and its alloy in KOH without and with CO_2_ were evaluated to validate the findings obtained from the potentiodynamic polarization. Figure 6 (a) shows a Nyquist plot for Zn and its alloy anodes in KOH electrolyte without CO_2_ at the *E*_corr_. The two investigated electrodes have high-frequency semicircles and low-frequency inclined lines. The diameter of the high-frequency semicircle quantitatively represents the obstacle to charge transfer between the electrolyte and electrode, whereas the low-frequency inclined line is caused by ion diffusion within the electrode^50^. Figure 6 (b) displays the impedance magnitude versus frequency Bode diagram, while Fig. 6 (c) presents the phase angle-frequency relationship of Zn and Zn-Bi electrodes in CO₂-free solution. These Bode plots enable the extraction of electrochemical parameters listed in Table 5. The derived parameters encompass solution resistance (R_s_), polarization resistance (R_p_), charge transfer resistance (R_ct_), and capacitance of the double-layer (C_dl_). Compared to its created alloy with bismuth, the diameter of the semicircle of the complex plan for pure Zn is smaller, as illustrated in Fig. 6 (a). This reveals that for the alloy, the behaviour of EIS grows more capacitive. This demonstrated how the resistance to corrosion of Zn was improved by the slight addition of bismuth. A tiny amount of bismuth added to zinc has been demonstrated to enhance the diameter of the EIS semicircle. For pristine Zn and Zn-Bi electrodes in the solution without CO_2_, the ions diffusion from the anode surface to the bulk of the electrolyte is responsible for the observed Warburg behavior (Z_w_)^33,49^. Electrochemical processes like the evolution of hydrogen and the solubility of Zn may be the cause of this tendency. However, it is noted that Fig. 6 (d) does not show the Warburg tail of the two anodes being studied when CO_2_ is present at low frequency. These findings suggest a substantial reduction in ionic diffusion processes. Consequently, CO₂ presence in the electrolyte effectively suppresses Warburg impedance characteristics. Furthermore, bismuth incorporation into zinc matrices increases charge transfer resistance (R_ct_) values. The data confirm that the bismuth-modified electrode exhibits superior corrosion resistance compared to unalloyed zinc in alkaline media. Based on the previous data, this pattern, which indicates the start of the corrosion, is consistent with the Tafel polarization portion. At the active region the alloy Nyquist plots exhibit semicircle behavior as a result, suggesting kinetic control of the electrochemical reaction. This further demonstrates how these outcomes align with the results of the Tafel graph.

Figure 6 (d) reveals that CO₂ introduction into KOH electrolyte produces substantially increased semicircle radii for both zinc and the zinc-bismuth alloy relative to CO₂-absent conditions, confirming enhanced corrosion protection at these conditions. Charge transfer resistance (R_ct_) measurements yield 100.507 and 182.881 Ω.cm² for zinc and its alloy electrodes, respectively. These impedance results corroborate the polarization data. Figure 6 (e, f) displays Bode magnitude and phase responses for zinc and Zn-Bi under CO₂ conditions. Warburg impedance (Z_w_) appears in both anodes during CO₂-free testing, attributed to mass transport limitations governing species migration from the electrode toward the bulk solution. This behavior can be linked to the start of corrosive processes, such as the evolution of hydrogen and the dissolution of zinc, and it is compatible with the Tafel polarization component. In contrast, it seems that the Warburg tail for Zn and Zn-Bi disappear during CO₂-free testing. This suggests that the carbonate compounds on the surface significantly reduce the diffusion of the ions. This led to the observation of sizable semicircle Nyquist plots at the active region of the two anodes under study. This behavior demonstrates how kinetic reactions govern electrochemical processes^31^. The analogous circuits that are suggested for fitting the acquired impedance data in the electrolyte under investigation in the absence and presence of CO_2_ are shown in Fig. 6 (g, h). Based on the fitted impedance data for two electrodes (Fig. 6), double-layer capacitance (C_dl_) and charge transfer resistance (R_ct_) values are calculated and displayed in Table 5. Double-layer capacitance (C_dl_) determination employs the relationship below:

13 $$\:f\left(-{Z}_{max}^{\prime \prime}\right)=\frac{1}{2\pi\:{C}_{dl}{R}_{ct}}\:\:\:\:\:\:\:\:\:\:\:\:\:\:\:\:\:\:\:\:\:\:\:\:\:\:\:\:\:\:\:\:\:\:\:\:\:\:\:\:\:\:\:\:\:\:\:\:\:\:\:\:\:\:\:\:\:\:\:\:\:\:\:\:\:\:\:\:\:\:\:\:\:\:\:\:\:\:\:\:\:\:\:\:\:\:\:\:\:\:\:\:\:\:\:\:\:\:\:\:\:\:\:\:\:\:\:\:\:\:\:\:\:\:\:\:\:\:\:\:\:\:\:\:\:\:\:\:\:$$

Here (- Z”_max_) is the maximal imaginary component of the impedance.

The C_dl_ is shown to decrease in the investigated electrolyte in the absence and presence of CO_2_ as the R_ct_ rises. When an ionic liquid is present, a drop in (C_dl_) values implies that ionic liquid molecules are replacing the water molecules. This decrease can be ascribed to either a minimum in the local dielectric constant or a rise in the thickness of the electrical double layer^51,52^.

**Table 5:** EIS parameters for Zn and Zn-Bi in KOH electrolyte under CO₂-free and CO₂-containing conditions at 25 °C.

Electrolytes	Electrodes	Parameters			
		*R*_s_ (Ω.cm^2^)	*R*_*p*_ (Ω.cm^2^)	*R*_ct_ (Ω.cm^2^)	C_dl_ (F.cm^2^)
**25 °C**					
KOH	Zn	6.5372	37.102	30.5648	1.65 × 10^− 4^
	Zn-Bi	3.7947	55.311	51.5163	4.90 × 10^− 5^
KOH + CO_2_	Zn	11.343	111.85	100.507	2.51 × 10^− 5^
	Zn-Bi	10.199	193.08	182.881	1.74 × 10^− 5^

###  Charge-discharge process

**Fig. 7:**
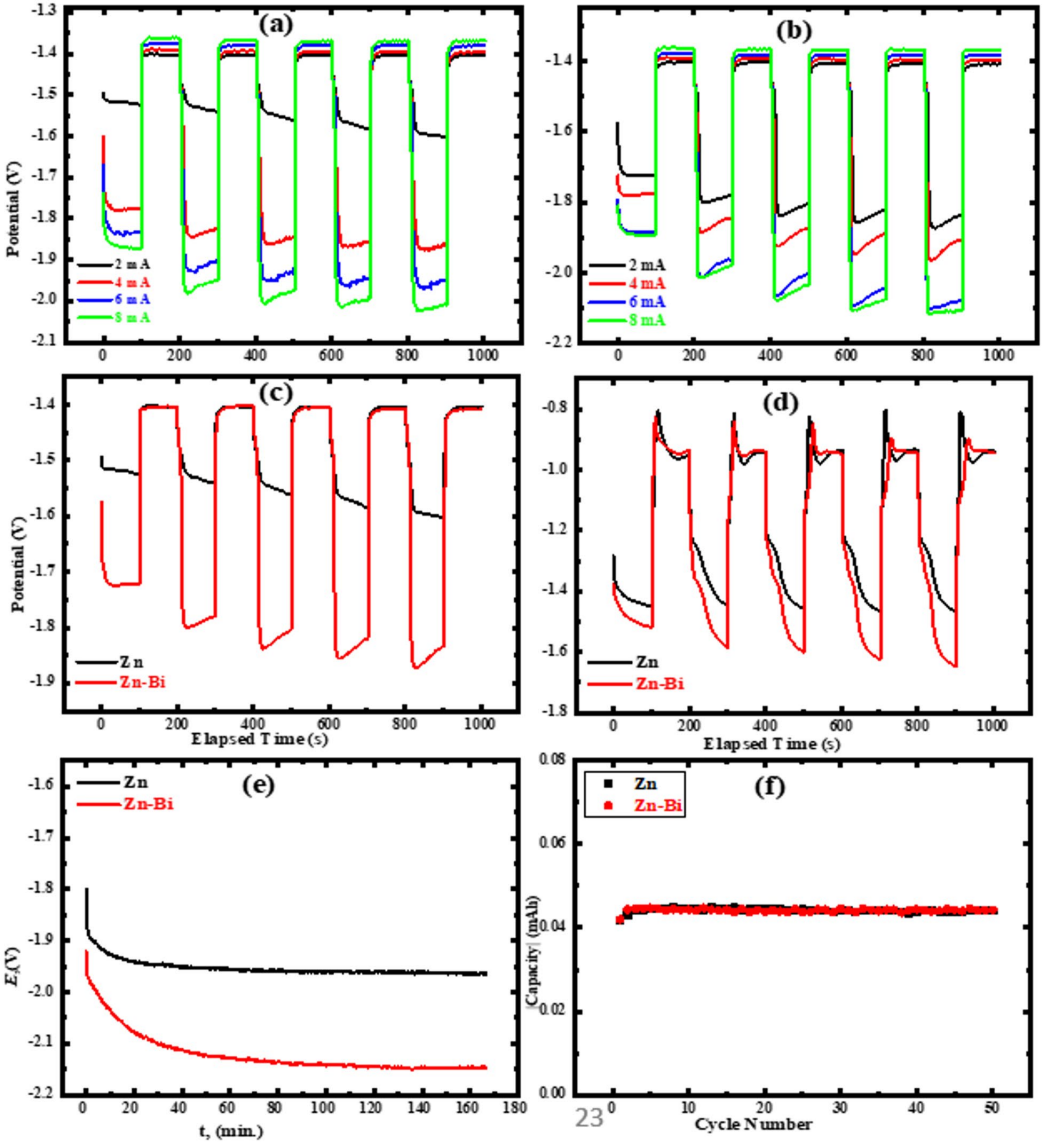
The galvanostatic cycling plots at 25 °C for Zn (**a**) and Zn-Bi (**b**) electrodes under varying current density conditions. (**c, d**) Galvanostatic cycling behavior of zinc and its alloy at ± 2 mA/cm² current density in KOH without and with CO₂, respectively. The long-term behaviour of Zn and Zn-Bi at -8 mA/cm² (**e**) and stability of cycling (**f**).

The effectiveness of Zn and Zn-Bi anodes in galvanostatic charge-discharge was assessed to assess their suitability as electrodes in alkaline battery systems in KOH electrolyte under various scenarios. Figure 7 (a, b) shows the charge-discharge galvanostatic technique for Zn and Zn-Bi in KOH without carbon dioxide at different current densities (± 2, ± 4, ±6, and ± 8). The figure illustrates that when the applied current density grows from ± 2 to ± 8 mA cm^− 2^. The charge-discharge potential difference of the previously described electrodes also rises. This demonstrates that the electrodes being studied possess favourable charge-discharge characteristics, and a satisfactory discharge level was attained at several applied current densities. But in line with other research, an increase in current density also results in a shorter discharge duration^31^, indicating a process that is diffusion-limited at greater rates. Figure 7 (c) displays the charge-discharge plots for Zn and its alloy electrodes in KOH without CO_2_ at an applied current of ± 2 mA/cm^2^. When compared to zinc, the alloy exhibits the largest potential difference and discharge time, demonstrating decreased internal polarization and increased electrochemical reversibility. This enhancement can be explained by bismuth’s ability to suppress unwanted reactions, especially the hydrogen evolution process (HER), which delays self-discharge and raises the electrode’s total discharge efficiency. The improved performance is consistent with the critical role that zinc anode behavior plays in influencing the stability and energy density of zinc-based alkaline batteries^46^. The performance of zinc electrodes in alkaline media is widely acknowledged as the primary factor governing energy density in rechargeable zinc-air battery systems^19^. The hydrogen naturally evolves on the surface of the Zn electrode in an alkaline electrolyte; however, in certain situations, the zinc oxidation may not be suitable for the zinc electrode. Consequently, an improvement in discharge efficiency results from the alloying of bismuth with zinc, which delays the zinc self-discharge spontaneously. Zinc and Zn-Bi electrodes’ charge-discharge galvanostatic patterns in KOH with carbon dioxide at a current of ± 2 mA/cm^2^ (at 25 °C) are illustrated in Fig. 7 (d). It is clear that the alloy exhibits a greater potential difference and discharge time than pure zinc when CO_2_ is present. Additionally, a greater potential fluctuation and longer discharge time are shown when comparing the absence and presence of carbon dioxide gas. This suggests that CO_2_ synergistically improves electrode-electrolyte interface stability, perhaps as a result of protective carbonate coatings that reduce HER and corrosion. Furthermore, more complicated interactions on the surface that ultimately improve performance enhancement are suggested by the wider potential fluctuation seen in CO_2_-saturated environments. At a high current density of -8 mA/cm^2^, the long-term discharge behavior and cycling stability of zinc and its alloy electrodes are shown in Fig. 7 (e, f). The long-term discharge of the investigated electrodes reveals distinct electrochemical stability windows. The pure Zn electrode exhibits stable discharge performance down to -1.95 V, whereas the Zn alloy electrode maintains stability at a more negative potential of -2.15 V. This difference reflects the influence of alloying on the electrochemical kinetics and interfacial reactions, including modified zinc dissolution behavior and suppression of parasitic side reactions. Consequently, electrode-specific lower cut-off discharge potentials were applied during galvanostatic cycling to ensure operation within the intrinsic stability range of each electrode. This approach prevents over-discharge, minimizes accelerated corrosion or hydrogen evolution, and enables a fair and realistic assessment of long-term cycling durability. Bismuth alloying has a positive effect on reducing zinc corrosion and hydrogen gas development, as demonstrated by the improved capacitance and stability of the Zn-Bi electrode.

Generally. The results demonstrate that the electrochemical behavior of Zn electrodes in alkaline conditions is greatly improved by modest alloying with bismuth, which inhibits parasitic processes and increases electrode activity. As a result, the Zn-Bi alloy exhibits increased resistance to deterioration, longer cycle life, and better discharge capacity, making it a viable option for cutting-edge rechargeable alkaline battery technologies.

**Fig. 8:**
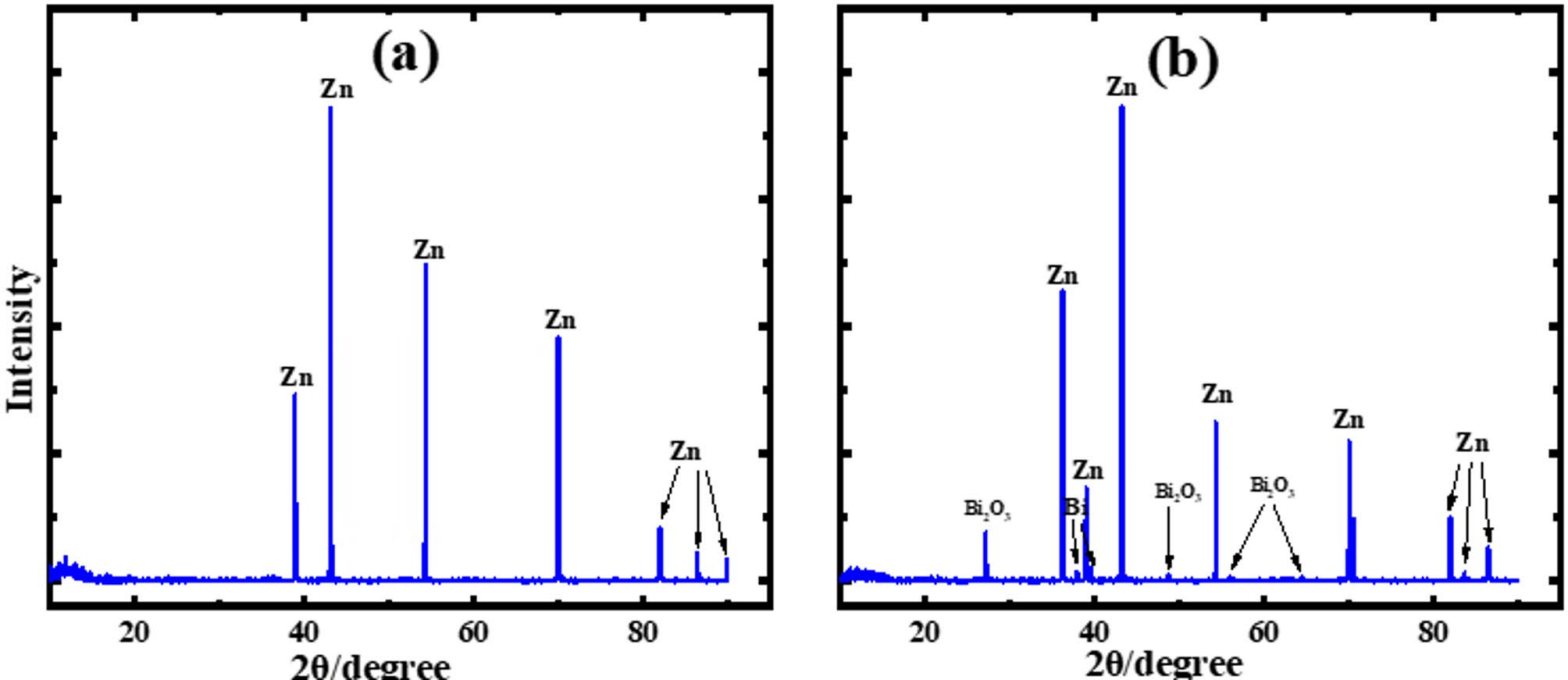
XRD Electrochemical products generated on the zinc surfaces (**a**) and Zn-Bi (**b**), at 25 °C in KOH after cycling at the region of activity, respectively.

Figure 8 shows the XRD patterns of the pure Zn and Zn–Bi anodes after electrochemical cycling in alkaline media. For the pure Zn electrode, the diffraction pattern after cycling exhibits noticeable changes in peak intensities, along with the appearance of additional weak reflections associated with surface oxidation and corrosion-related phases. These changes indicate partial structural degradation of the zinc surface during prolonged cycling, which is consistent with the pronounced corrosion activity and parasitic hydrogen evolution typically observed for pure zinc in alkaline electrolytes.

In contrast, the Zn–Bi anode displays significantly improved structural stability after cycling. The post-cycling XRD pattern of the Zn–Bi electrode retains the dominant diffraction peaks of metallic zinc without peak disappearance or severe broadening, indicating preservation of the crystalline framework. The absence of pronounced new peaks related to bulk corrosion products suggests that bismuth incorporation effectively mitigates extensive phase transformation during cycling.

The enhanced structural integrity of the Zn–Bi anode can be attributed to the synergistic effect of bismuth alloying and surface passivation, which suppresses zinc dissolution and limits the formation of detrimental corrosion phases. Minor variations in peak intensity are likely associated with the formation of thin surface layers rather than bulk structural changes. These observations are in good agreement with the electrochemical results, which demonstrate reduced corrosion current density, suppressed hydrogen evolution, and improved charge–discharge stability for the Zn–Bi system.

## Conclusions

This comprehensive investigation systematically evaluated the corrosion characteristics and the electrochemical performance of bismuth-modified zinc electrodes for alkaline battery applications, employing multiple characterization techniques, including galvanostatic cycling, electrochemical impedance spectroscopy (EIS), and potentiodynamic polarization analysis. The research demonstrates that trace bismuth incorporation (0.5 wt%) fundamentally transforms zinc electrode behavior through multiple synergistic mechanisms that directly address critical challenges in alkaline power systems. Potentiodynamic polarization measurements revealed that Zn-0.5%Bi alloys exhibit substantially reduced anodic and cathodic current densities compared to pure zinc, indicating enhanced kinetic stability and reduced parasitic reactions. The introduction of CO₂ into the alkaline electrolyte produced remarkable performance enhancements, shifting corrosion potentials toward more noble values while dramatically reducing corrosion current densities from 99.311 to 26.92 µA cm⁻² for zinc and from 41.399 to 12.3 µA cm⁻² for the Zn-Bi alloy. This exceptional corrosion inhibition efficiency of 87.6% for the alloy under CO₂-saturated conditions stems from the formation of protective zinc carbonate (ZnCO₃) surface films, which effectively passivate the electrode interface and suppress the detrimental hydrogen evolution reaction that typically limits zinc anode performance. Electrochemical impedance spectroscopy validated these findings, demonstrating that bismuth alloying significantly increases charge transfer resistance (R_ct_) values while reducing double-layer capacitance (Cdl), indicating enhanced electrode-electrolyte interface stability. The disappearance of Warburg impedance behavior in CO₂-containing electrolytes confirms the formation of protective surface layers that minimize ion diffusion limitations and improve charge transfer kinetics. Galvanostatic charge-discharge evaluation revealed superior battery performance characteristics for the Zn-Bi system, including extended discharge durations, enhanced capacity retention, and improved cycling stability across multiple current densities. The alloy’s ability to maintain stable potential profiles during extended cycling directly translates to enhanced energy efficiency and extended operational lifetime for alkaline battery systems, addressing key commercial viability concerns. Surface characterization through XRD, EDS, and SEM analyses elucidated the mechanistic basis for performance improvements. In CO₂-free conditions, the alloy surface develops Bi₂O₃ and ZnO phases that provide moderate protection. However, CO₂ introduction facilitates ZnCO₃ formation, creating highly protective, adherent surface films that dramatically suppress both corrosion and hydrogen evolution. This CO₂-responsive surface chemistry represents a paradigm shift, transforming typically detrimental atmospheric CO₂ into a performance-enhancing component. The exceptional electrochemical stability observed for Zn-Bi alloys under CO₂-rich conditions has significant implications for practical battery deployment, particularly in ambient air-exposed systems where atmospheric CO₂ interaction is unavoidable. The research demonstrates that strategic micro-alloying can transform environmental challenges into performance advantages, offering a viable pathway for developing more robust and efficient alkaline energy storage systems. These findings establish bismuth-modified zinc anodes as highly promising candidates for next-generation alkaline battery technologies, offering simultaneous improvements in corrosion resistance, hydrogen evolution suppression, and electrochemical durability. The synergistic effects of bismuth alloying and CO₂-induced surface passivation provide a comprehensive solution to longstanding zinc anode limitations, positioning this material system for commercial implementation in advanced alkaline power sources with superior operational reliability and extended service life.

## Data Availability

All datasets used and/or analyzed in this study are available from the corresponding author upon reasonable request.
